# Thermal Reactivity of Bio-Oil Produced from Catalytic Fast Pyrolysis of Biomass

**DOI:** 10.1021/acs.energyfuels.4c03430

**Published:** 2024-10-01

**Authors:** Steven M. Rowland, Rianna Martinez, Cody J. Wrasman, Kristiina Iisa, Mark R. Nimlos, Michael B. Griffin

**Affiliations:** National Renewable Energy Laboratory, 15013 Denver West Parkway, Golden, Colorado 80401, United States

## Abstract

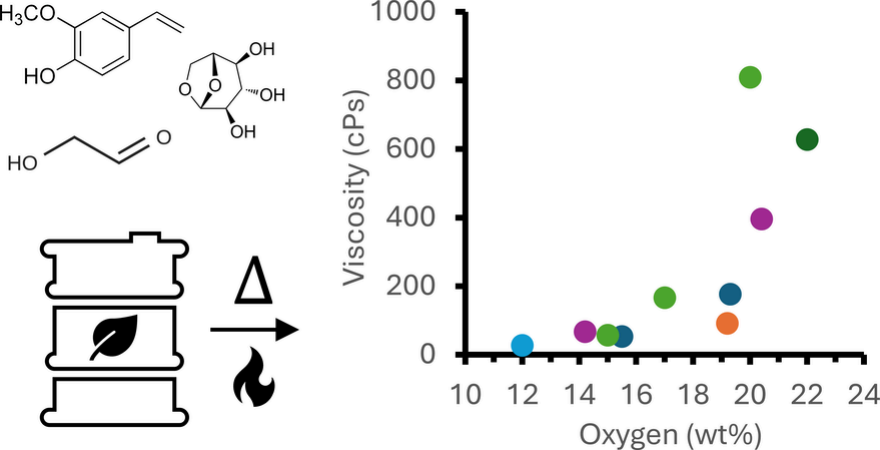

Catalytic fast pyrolysis (CFP) of biomass is a versatile thermochemical process for producing a biogenic oil that can be further upgraded to sustainable transportation fuels, chemicals, and materials. CFP oil exhibits reduced oxygen content and improved thermal stability compared to noncatalytic fast pyrolysis oil. However, some level of reactive oxygenates remain in CFP oils, and reactions between these species can result in molecular weight growth and increased viscosity, leading to the potential for challenges during transportation, storage, and downstream processing. Previous research has provided considerable insight into the reactivity of noncatalytic fast pyrolysis oils, but CFP oils have yet to be studied in a similar fashion. Consequently, the degree of catalytic upgrading that is necessary to effectively stabilize CFP oils has yet to be established, and little is known about the mechanistic details underlying the process. The current study addresses this knowledge gap by controlling the CFP reaction conditions to systematically vary the oxygen content of the resulting oil. Accelerated thermal reactivity studies were then performed, and the CFP oils were analyzed using gas chromatography–mass spectrometry (GC-MS), Fourier transform ion cyclotron mass spectrometry (FT-ICR MS), gel permeation chromatography (GPC), and viscometry to evaluate the impact of heating on their physical and chemical properties. The results revealed that short chain carbonyls, anhydrosugars, and lignin derivatives with conjugated vinyl groups likely play a role in the thermal reactivity of CFP oils. Additionally, experiments performed across a wide variety of feedstocks revealed relatively low thermal reactivity for CFP oils with oxygen contents of <20 wt %. However, above this threshold value, the thermal reactivity grew exponentially as a function of oxygen content, resulting in large increases in viscosity and molecular weight. These results serve to deepen the mechanistic understanding of CFP oil thermal reactivity and help inform the development of quality specifications for catalytic upgrading to effectively stabilize CFP oils.

## Introduction

Fast pyrolysis (FP) is a versatile technology pathway for the conversion of biomass and waste carbon into sustainable fuels, chemicals, and materials. Biomass FP is compatible with a variety of feedstocks, occurs under moderate conditions, and has been demonstrated on the commercial scale. However, the widescale deployment of biomass FP has been hindered by the low quality of the bio-oil it produces.^1−5^ Noncatalytic FP oils have highly oxygenated organic compounds that are entrained with water to form a stable suspension. The organic components largely consist of small acids (e.g., acetic acid), anhydrosugars (e.g., levoglucosan), small carbonyls (e.g., hydroxyacetaldehyde), and other oxygenates (e.g., furans, phenols, and lignin-derived compounds). The oxygen content of FP oils commonly ranges from 35 to 45 wt %,^1,6,7^ and the overall concentration of known reactive compounds (e.g., levoglucosan, hydroxyacetaldehyde, acetic acid, and hydroxypropanone) can comprise >20% of the sample.^7^ Specifically, the high reactivity and thermal instability of FP oil increase risks associated with transportation, storage, and downstream processing. The thermal stability and aging of FP oils have been widely studied. Oasmaa et al. investigated the effects of storage on FP oil composition and physical properties.^8^ In this previous research, the concentration of carbonyls was used to evaluate stability since these compounds are typically consumed during aging, and both the temperature and storage time affected the stability of FP oil. The carbonyl content was shown to be relatively stable for 4 years at −16.5 °C but decreased substantially at 9 and 22 °C. Further, the decrease in carbonyl content was correlated with an increase in viscosity and water insoluble lignin compounds. Similar results were reported by Cai and co-workers, who identified a >50× increase in viscosity after FP oil storage at 25 °C for 2 years.^9^ In other work by Oasmaa and Kuoppala, the amount of high- and low-molecular-weight lignin was shown to increase with storage time for up to 12 months under ambient conditions as well as during accelerated aging at 80 °C for 24 h.^3^ The underlying chemistry of FP reactivity was further investigated by Joseph et al., who showed the consumption of a wide range of pyrolysis compounds during accelerated aging.^10^ The reactive compounds identified include small aldehydes, vinyl-containing lignin monomers, di- and trihydroxybenzenes, and monosaccharides. This work also showed that the concentration of low- and high-molecular-weight lignin, as well as other high-molecular-weight compounds, increased substantially upon heating at 80 °C. The group proposed several reaction mechanisms that could lead to the formation of high-molecular-weight compounds, which included the stabilization of quinone methides from vinyl-containing lignin derivatives that can undergo nucleophilic addition with reactive groups within pyrolysis oil. It has also been shown that reactions between small aldehydes and phenols in the presence of acid can lead to the formation of large polymers, and this chemistry is the basis of phenol/formaldehyde resins such as novolaks or resols. Coupling reactions such as these lead to molecular weight growth and increased viscosity, which can result in phase separation and gum formation that negatively impact downstream processing, such as hydrotreating.^5,10−12^ These challenges are exemplified by reports of rapid plugging during the hydroprocessing of FP oils,^13^ which necessitates a two-stage process that includes an initial low-temperature stabilization step.^13−15^ The rapid coke formation is attributed to unsaturated bonds in carbonyls and carbon–carbon double bonds in, for example, lignin side chains.^15^ To combat reactive polymerization, a stabilization step at temperatures below 300 °C can be introduced to hydrogenate reactive components and increase the H/C ratio while the O/C ratio remains relatively constant.^15,16^

One strategy to improve bio-oil quality is to catalytically upgrade the pyrolysis vapors prior to condensation.^17^ This approach, which is generally referred to as catalytic fast pyrolysis (CFP), has been shown to increase bio-oil heating value, reduce acidity, and improve stability compared to noncatalytic FP oil.^17−22^ CFP also provides the ability to control the oxygen content and the overall quality of oil produced by the type of catalyst used and by the biomass-to-catalyst ratio selected,^20,23^ with most reported CFP oils ranging from 10 to 30 wt % oxygen.^24^ The enhanced stability of CFP oil is generally attributed to a reduction in the concentration of reactive oxygenates and has the potential to overcome important barriers associated with transportation, storage, and downstream processing. For example, Agblevor et al. reported a greater than 6-fold improvement in stability as measured by viscosity increase during storage of CFP oil vs FP oil.^25^ Likewise, previous studies have shown potential for single-stage hydroprocessing of CFP oils without requiring a low-temperature stabilization step.^21,26,27^ In one study, CFP oil with 22.5 wt % oxygen on a dry basis was successfully hydrotreated for over 300 h in a single-stage process without significant catalyst deactivation or coke formation.^26^ However, other studies have reported relatively rapid plugging or catalyst fouling during hydrotreating of CFP oil.^28^ For example, in one case, hydrotreating CFP oil with 19.5 wt % oxygen led to plugging in only 17 h. In other cases, longer durations were achieved, but the catalysts exhibited considerable deactivation over the course of the experiments.^29^ The limited amount of previous research focused on the thermal reactivity of CFP oils makes it difficult to reconcile these discrepancies, and although previous work on FP oils provides some context, the observations cannot be directly extended to CFP oils due to their distinct composition and properties. Consequently, the role of specific compound classes (e.g., aldehydes, acids, phenols) remains uncertain, and the required degree of catalytic upgrading to effectively stabilize the CFP oils has yet to be established.

The research presented here addresses these data gaps by evaluating the thermal stability of three CFP oils produced from the same biomass feedstock with varied degrees of catalytic upgrading, which resulted in the production of CFP oils with oxygen content ranging from 15 to 20 wt %. An accelerated aging method (heating to 100 °C for 0, 6, and 24 h) was applied to determine the reactivity of each oil. Gas chromatography–mass spectrometry (GC-MS) analysis was conducted to determine the chemical composition of small reactive compounds and their relative loss due to reactions during aging. Fourier transform ion cyclotron mass spectrometry (FT-ICR MS) was used to determine the chemical composition and reactivity of high-molecular-weight compounds as well as the products that were formed during aging, which were not detected by GC-MS. Gel permeation chromatography (GPC) analysis provided insight into the effect of catalytic upgrading and accelerated aging on the molecular weight distribution of the CFP oils, and all data were correlated with viscosity measurements to better understand the effect of chemical reactions that occur upon heating of CFP oil and the products that are formed, which may lead to downstream fouling. This approach provides useful insight into determining the types of molecules that contribute to the thermal reactivity of CFP oil and helps elucidate the underlying reaction mechanisms that occur during aging.

## Methods

### Fast Pyrolysis and Catalytic Fast Pyrolysis

CFP oils were produced using an *ex situ* reactor configuration described elsewhere.^27,30^ Feedstocks were first pyrolyzed at 500 °C in a 43 cm tall bubbling fluidized bed reactor with an internal diameter of 5.2 cm, using quartz sand as a bed material. Feedstocks were introduced into the system by using a K-Tron loss-in-weight feeder system. The bed was fluidized using 17.6 sl/min N_2_ at a pressure slightly above atmospheric. Char was removed from the pyrolysis stream using a cyclone, and the resulting vapors were upgraded in a second 5.2 cm diameter, 15 cm tall bubbling fluidized bed with a 7.8 cm diameter, 36 cm tall disengagement section that was continuously fed with a ZSM-5 catalyst provided by Johnson Matthey using a second K-Tron loss-in-weight feeder and held at a temperature between 500 and 525 °C. The upgrading reactor effluent was passed through a 2 μm stainless steel mesh hot gas filter to remove catalyst fines before entering a condensation train consisting of a jacketed chilled condenser held at −2 °C, an electrostatic precipitator, three dry ice condensers, and an ice-cooled coalescing filter. The gas stream exiting the condensation train was monitored using a moisture sensor, a dry test meter, an online Agilent 490 micro GC instrument for CO_2_, CO, H_2_, and C_1_–C_4_ hydrocarbon quantification, a nondispersive infrared analyzer (Model 300 from California Analytical Instruments), and an online Agilent 7890B GC-MS instrument connected to a 5977A MSD (mass selective detector) and FID (flame ionization detection) for condensable gas quantification. All parts between the pyrolyzer and the condensation system were maintained at between 400 and 500 °C by using electric heating tapes. The biomass-to-catalyst ratio was varied by changing the catalyst feed rate to attain ratios between 1.5 and 3.0. All experiments were performed until either 1800 g of biomass was fed or a system pressure drop of 60 kPa was reached due to system fouling. The product liquids from the jacketed condenser, the electrostatic precipitator, and the dry ice condensers were combined and allowed to phase separate into oil and aqueous fractions. This work focuses on the composition and stability of the oil fractions. Several different feedstocks comprising pine and pine forest residue fractions were used, and the ultimate analysis for each is given in Table S1. For the 50:50 blend of clean pine wood and forest residues, a series of experiments with different biomass:catalyst (B:C) ratios were performed. Previous CFP experiments performed using the same reactor system and catalyst formulation have demonstrated good run-to-run reproducibility, with standard deviations on oil mass yield and oxygen content ranging from ±0.3–0.9 wt % and ±0.6–1.3 wt %, respectively.^22^ Select CFP oils were evaluated via acid titrations and inductively coupled plasma optical emission spectroscopy (ICP-OES). Carboxylic acid number was determined by a modified ASTM D7544 method.^31,32^ Noncatalytic fast pyrolysis of a pine forest residue feedstock was performed in the same reactor unit described above. The feedstock was fed at 300 g/h, and pyrolysis was conducted at 500 °C. The catalytic upgrading bed was bypassed, and the oils were collected in a condensation system. No phase separation was observed, and the thermal stability of the condensed product was measured in its entirety.

### Accelerated Aging

CFP oils were added to glass vials (3–7 g) and sealed to prevent loss of volatiles. The vials were heated to 100 °C for fixed time intervals (0, 6, and 24 h). The pre-reaction oils and thermally aged oils were analyzed by GC-MS, GPC, FT-ICR MS, and viscometry, as described below.

### Gas Chromatography with Mass Spectrometry (GC-MS)

Gas chromatography with mass spectrometry and flame ionization detection (GC/MS-FID-Polyarc) was used to measure volatile and semivolatile components. This analysis is similar to NREL LAP NREL/TP-5100-65889, Quantification of Semi-Volatile Oxygenated Components of Pyrolysis Bio-Oil by Gas Chromatography/Mass Spectrometry (GC/MS) but utilizes dual qualitative and quantitative detectors.^33^ The instrument utilized was an Agilent 8890 GC with a 5977B MSD. The GC was equipped with a post column flow splitter for simultaneous MS-FID analysis. A Polyarc converter (methanizer) was placed in line with the FID for the robust quantitation of oxygenated species. Samples were diluted ∼1:40 gravimetrically in acetone and injected in a volume of 1.0 μL with the inlet set to 250 °C and a split ratio of 20:1. The column used for separation was an Rtx-1701 (Restek, 14% cyanophenyl polydimethylsiloxane 60 m × 250 μm × 0.25 μm). The initial oven temperature was set to 45 °C and held for 10 min, followed by a ramp of 3 °C/min to 260 °C, which was held for 5 min. The MSD was operated in continuous scan mode from *m*/*z* 29 to 400 with a solvent delay time of 7.7 min. Both FID and MSD transfer lines were set to 350 °C. Neat standards of representative compounds were used to prepare a mixture for instrument calibration to verify the Polyarc response using an 8-point curve with a resultant *R*^2^ of ≥0.999. Relative standard deviations for compounds in the calibration mixture injected multiple times across an analysis set were ≤5%. Method accuracy determined using calibration standard verification was <5%.^33^

### Gel Permeation Chromatography (GPC)

The molecular weight distribution in the CFP oils were measured by gel permeation chromatography (GPC). Samples (50 mg) were dissolved in 50 mL of tetrahydrofuran (THF, no inhibitor). The dissolved samples were filtered (0.45 μm nylon membrane syringe filters) before GPC analysis. GPC analysis was performed using an Agilent HPLC instrument (1260 Infinity) with three GPC columns (Polymer Laboratories, 300 × 7.5 mm) packed with polystyrene-divinylbenzene copolymer gel (10 μm beads) having nominal pore diameters of 10^4^, 10^3^, and 10^2^ Å, respectively. The eluent was THF, and the flow rate was 1.0 mL/min. The sample concentration was 1–2 mg/mL, and an injection volume of 25 μL was used. The HPLC was attached to a diode-array detector measuring absorbance at 260 nm (bandwidth of 40 nm). Retention time was converted into molecular weight by applying a calibration curve established using 18 polystyrene standards of known molecular weight, ranging from 580 to 980 000 g/mol, and toluene was used to calibrate for low mass compounds (92 g/mol). The calculated molecular weights are not absolute molecular weights but are an approximation based on the polystyrene standards. The GPC data were normalized to the total response for each sample prior to calculations and plotting.

### Fourier Transform Ion Cyclotron Resonance Mass Spectrometry (FT-ICR MS)

All samples were analyzed by Fourier transform ion cyclotron resonance mass spectrometry (FT-ICR MS) with chemical ionization. The data were collected with a Bruker Solarix 7 T FT-ICR MS instrument. The samples were dissolved in acetone to 10 mg/mL and then further diluted in methanol to a final concentration of 150 μg/mL for analysis. The samples were then directly infused at a flow rate of 20 μL/min, and 25 individual spectra were co-added to produce the final averaged spectrum. Atmospheric pressure chemical ionization (APCI) was used based on previous work with the following conditions:^34^ capillary voltage, 4500 V; end plate offset, −500 V; nebulizing gas pressure, 2 bar; dry gas temperature, 200 °C; desolvation gas, 2.5 L/min; corona, 4500 nA; low *m*/*z* cutoff, *m*/*z* 100; and time-of-flight, 0.7 s. The data were exported to CSV, and chemical formula assignments were generated with PetroOrg software.

### Viscosity

Viscosity measurements were performed for 0, 6, and 24 h time points from the accelerated aging of CFP oil. The method used was based on ASTM D445-24.^35^ Measurements were taken at 40 °C using a Brookfield DB2T viscometer outfitted with a 40Z spindle.

## Results and Discussion

### Characterization of CFP and FP Oils

To better understand the reactions that occur in CFP oil during accelerated aging tests, the chemical composition of three CFP oils, with varied oxygen contents, was compared to a representative FP oil. To limit confounding variables, the CFP oils produced for this study were all from the same feedstock, a 50:50 blend of clean pine wood and forest residues. The extent of upgrading was varied by changing the biomass:catalyst ratio (B:C), which enabled a range of oxygen content from 15 to 20 wt %. Table 1 provides an overview of the feedstock information and process conditions used to generate the CFP oils.

**Table 1:** Select Feedstock Properties, Reaction Conditions, and Process Yields for CFP Oils

sample	feedstock	ash content, wt %	B:C ratio	oil organic oxygen content, wt % dry basis	oil organic fraction mass yield, wt % dry biomass basis	oil aqueous fraction mass yield, wt % dry biomass basis
CFP-15O	pine:forest residue blend	1.1	1.5	15	12	29
CFP-17O	pine:forest residue blend	1.1	1.7	17	14	29
CFP-20O	pine:forest residue blend	1.1	2.5	20	15	31

**Figure 1:**
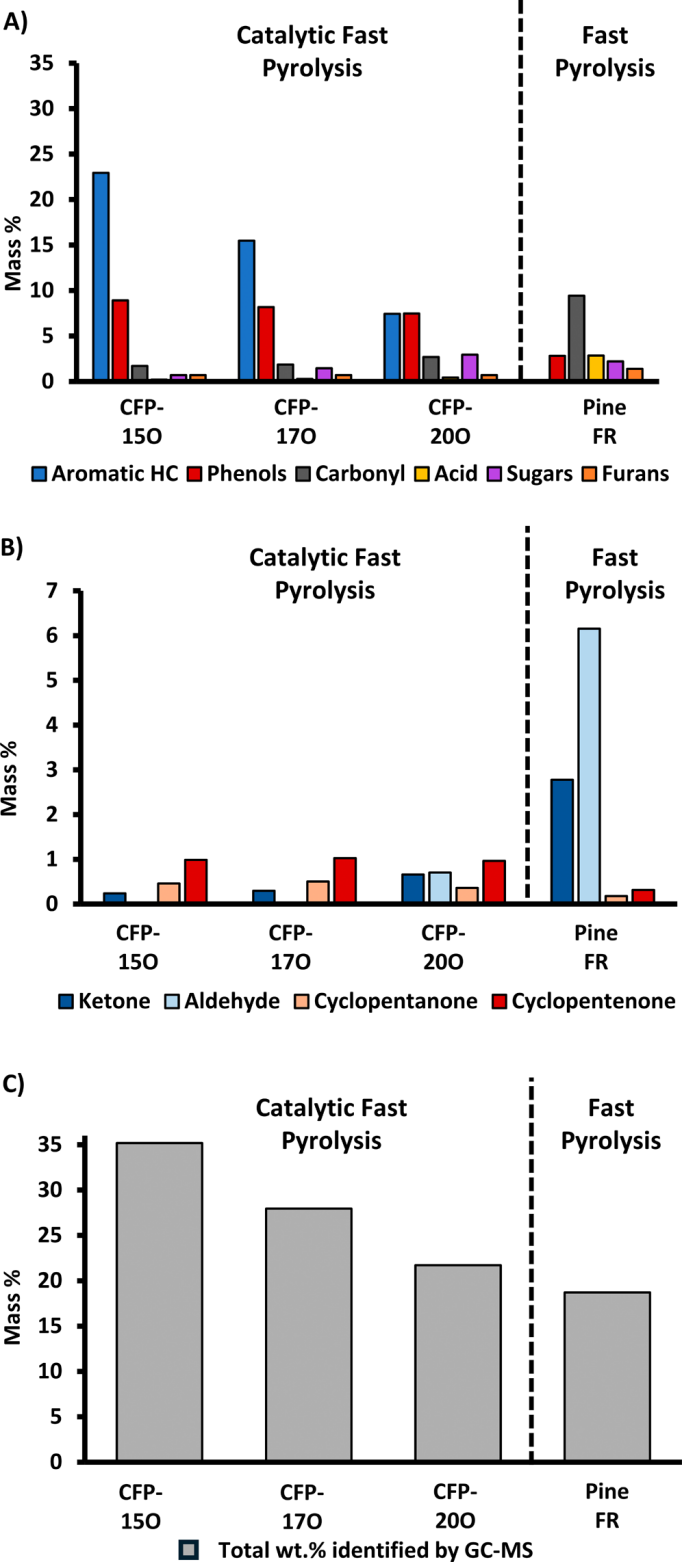
(A) Comparison between the composition of FP and CFP oils as measured by GC-MS. (B) Concentration of specific carbonyl compound groups measured in FP and CFP oils by GC-MS. (C) Total mass percentage of compounds detected by GC-MS. Molecules comprising each compound grouping are reported in Table S2.

Figure 1 highlights key differences in the chemical composition, observed by GC-MS, between CFP oils of varying oxygen concentrations and a representative FP oil with an oxygen content of 42 wt % on a dry basis. Figure 1A plots the GC-MS-derived chemical compositions grouped by compound categories. A full list of the compounds quantified in each category is provided in Table S2. In this work, CFP oils were produced with oxygen contents ranging from 15 to 20 wt %, which are referred to as CFP-15O, CFP-17O, and CFP-20O throughout the manuscript. During these experiments, the CFP oil naturally segregated into organic and aqueous fractions upon condensation, whereas the organic and aqueous fractions are typically contained within a single phase for noncatalytic pyrolysis oils. The separation of the aqueous phase during CFP serves to increase the energy density of the organic fraction, which was utilized for the stability experiments reported herein. The increase in oxygen content is primarily driven by higher concentrations of potentially reactive oxygenates such as acids, carbonyls, and anhydrosugars and is accompanied by a concomitant decrease in aromatic hydrocarbons. The overall mass of compounds observable by GC-MS also decreases with increasing oxygen content from 35% in CFP-15O to 22% in CFP-20O (Figure 1C). The decrease in the GC-MS detectable fraction indicates an increased concentration of higher-molecular-weight compounds, which may be unreacted pyrolysis products or oligomers formed during the condensation of reactive species. As expected, the total GC-MS detectable fraction for the FP oil (19%) is lower than that for the CFP oils, and the product composition differs considerably. The FP oil exhibits a complete absence of aromatic hydrocarbons and much lower concentrations of detected phenols. Conversely, the FP oil is enriched in carbonyls and acids, primarily acetic acid (2 wt %), in comparison to the CFP oils. As such, it is expected that all of the CFP oils explored in this study will exhibit reduced reactivity compared with FP oil even at the highest oxygen content. As shown in Figure 1B, the overall carbonyl content increases from 1.7% to 2.7% as the oxygen content increases from 15 to 20 wt %. This 59% relative increase in reactive carbonyls can accelerate cross-linking of larger molecules, resulting in increased viscosity and potentially leading to reactor plugging or other upsets in downstream processing. Aldehydes are a particularly reactive class of carbonyls since they are less sterically hindered than ketones and only have a single alkyl group to compensate for the partial positive charge on the carbonyl carbon. As shown in Figure 1B, aldehyde concentrations are below detection limits for CFP-15O and CFP-17O but increase to 0.7% for CFP-20O. In contrast to CFP oil, the detectable fraction of the FP oil contains carbonyl concentrations of 9.4%, with 6.2% of the sample being composed of aldehydes and 2.8% composed of linear ketones.

**Figure 2:**
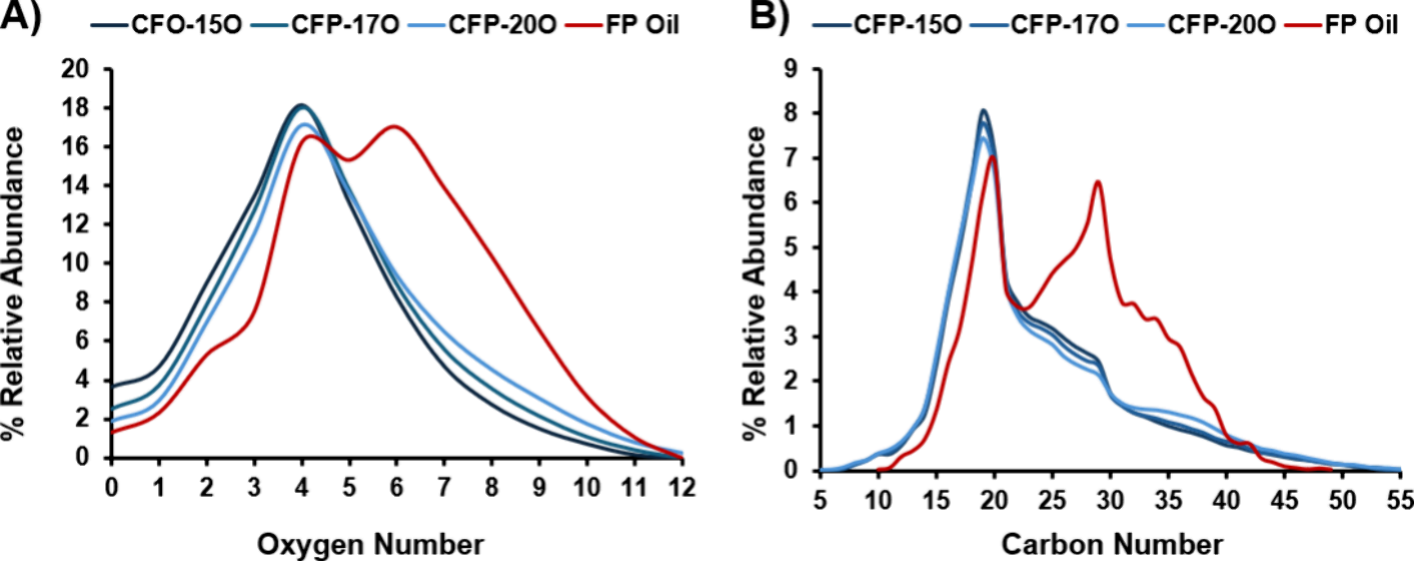
Positive-ion APCI FT-ICR MS data for (A) oxygen distribution and (B) carbon distribution for FP and CFP oils.

While the GC-MS data provide useful insight into the composition of the oils, it is important to note that only 22–35 wt % of the molecules were detectable using this technique. To complement these data, we also analyzed the CFP and FP oils by FT-ICR MS. FT-ICR MS provides access to the chemical composition for a wide range of compounds based on accurate mass measurements, along with ultrahigh resolving power, and has been widely used for the analysis of petroleum and environmental samples.^36,37^ This approach is particularly useful for gaining insight into the composition of the high-molecular-weight fraction (>200 Da), which is not detectable by GC-MS. Figure 2 shows the oxygen and carbon number distributions for the CFP and FP oils, as measured by FT-ICR MS. Figure 2A reveals that the CFP oil has an oxygen distribution with a single apex at 4 oxygen atoms per molecule. The FP oil has a larger number of oxygen atoms per molecule in a bimodal distribution with an apex at 4 and 6 oxygen atoms per molecule. The carbon distribution, shown in Figure 2B, shows a multimodal profile for the FP oil with prominent apexes observed at C_20_ and C_29_ and a smaller feature from C_32_–C_40_. The CFP oil shows a major apex at C_19_ with shoulders in two distinct ranges from C_22_ to C_30_ and from C_30_ to C_40_. These ranges for FP and CFP oils correspond with increases in approximately C_8–__10_ increments, which is consistent with the mass profiles of lignin-derived oligomers.^38^ Compared to FP oil, there are considerably fewer compounds above C_22_ in CFP oil. There is, however, a relatively higher percentage of compounds in CFP oil with a carbon number of >43 compared to FP oil.

**Figure 3:**
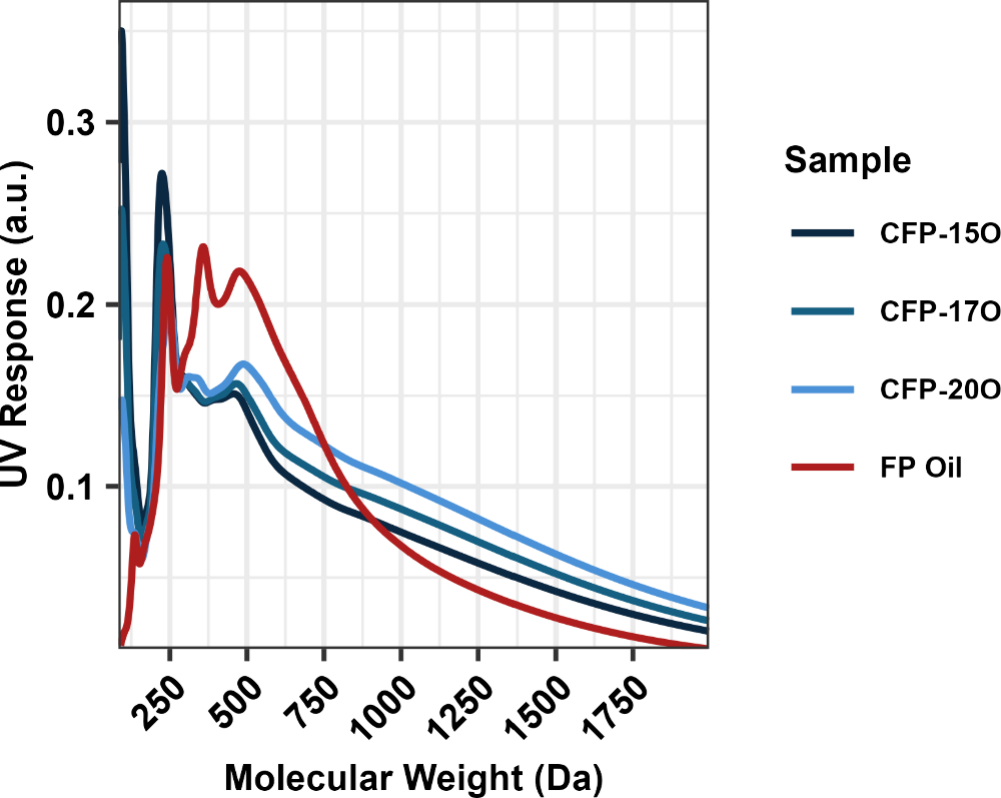
Molecular weight distribution as measured by GPC for CFP and FP oils.

To gain further insight into the molecular weight distribution, GPC was performed for the CFP and FP oils. It should be noted that GPC provides an apparent molecular weight based on calibrations using polystyrene standards, and the absolute molecular weights may be shifted from the reported values. Nonetheless, this technique provides a valuable qualitative assessment and can be used to compare the molecular weight distributions between samples in a self-consistent manner. The data in Figure 3 reveal a higher mass fraction in the region from 350 to 750 Da for FP oil when compared to CFP oil. This region has been reported to correspond to pyrolytic lignin in both CFP and FP oils.^39,40^ The data suggest that pyrolytic lignin may be catalytically deconstructed during CFP to form smaller compounds compared to noncatalytic FP oil. Interestingly, the CFP oils also showed an increase in the relative proportion of sample with an apparent molecular weight of >1000 Da. This observation is consistent with the FT-ICR MS data, which revealed a higher relative abundance of compounds with >43 carbon atoms per molecule in CFP oil compared to FP oil. The extent of catalytic upgrading (i.e., oxygen content) was shown to correlate with the molecular weight distribution of the CFP oils, where CFP oils with higher oxygen content had lower relative amounts of compounds <300 Da and higher relative amounts of compounds >500 Da. The high-molecular-weight compounds may be formed via breakthrough of unreacted pyrolysis vapors at higher B:C ratios or coupling of reactive monomers either in the gas phase or after condensation to from larger oligomers. The latter hypothesis is supported by previous work, which showed that pyrolysis of lignin monomer model compounds produces a condensed oil with polymers of up to 6 subunits.^41^ Regardless of the mechanism, it is apparent that the degree of catalytic upgrading serves as an effective mechanism to tailor the oxygen content and molecular weight distribution of the CFP oil.

### Thermal Stability of CFP Oils

**Figure 4:**
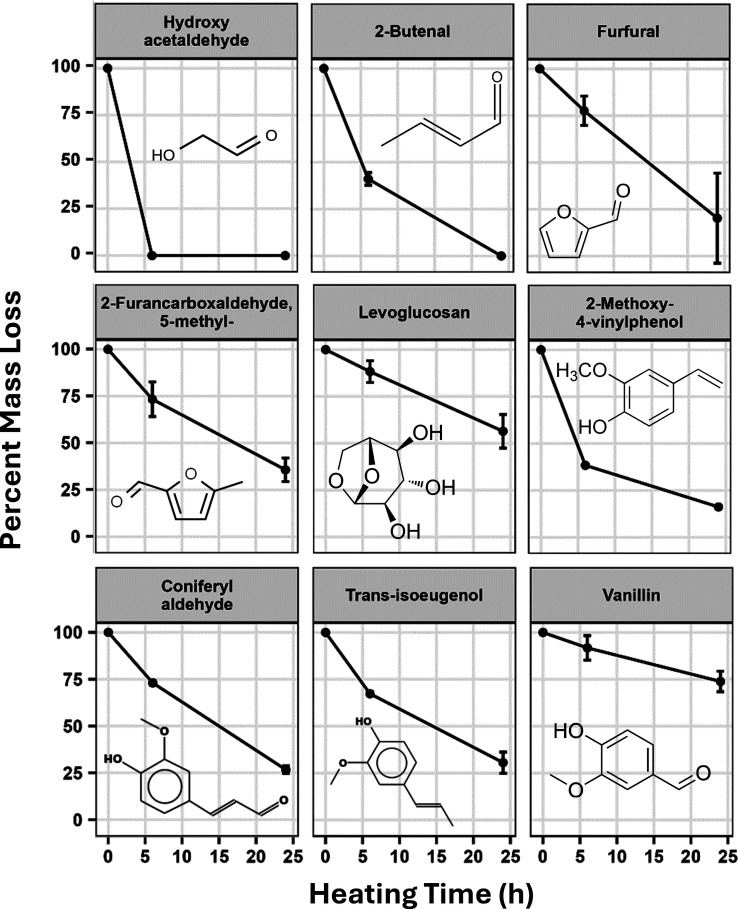
Percentage of initial concentration plotted vs heating time for select compounds during accelerated aging experiments as measured by GC-MS. The data represent cumulative averages taken from measurements of CFP-15O, CFP-17O, and CFP-20O.

Accelerated aging experiments were performed to evaluate the changes in the chemical composition and physical properties of CFP oil upon heating. These experiments were performed at 100 °C, and samples were collected at 0, 6, and 24 h. Each sample was analyzed via GC-MS, FT-ICR MS, GPC, and viscometry to provide complementary insight into the physical and chemical transformations within the oils. GC-MS analysis identified 144 unique compounds, and the consumption during heating for the most reactive species is plotted in Figure 4. The data are reported as a percentage of the initial value and represent the average taken from CFP-15O, CFP-17O, and CFP-20O. The concentration of hydroxyacetaldehyde (HAA) exhibited a rapid decrease to below detection limits within 6 h of heating. The concentrations of other aldehydes, such as 2-butenal, furfural, and 5-methyl-2-furancarboxaldehyde, also declined but at a slower rate than HAA. The concentration of the anhydrosugar levoglucosan decreased by approximately 50% after 24 h of heating. Interestingly, significant reactivity was also observed for lignin derivatives such as 2-methoxy-4-vinylphenol, coniferyl aldehyde, and *trans*-isoeugenol. These lignin-derived compounds have conjugated vinyl groups that can result in resonance stabilized protonated quinone methide structures upon heating that are susceptible to nucleophilic addition by many functional groups present in CFP oils.^10^ Vanillin, a phenolic aldehyde with additional hydroxyl and ether functionality, also exhibited mild reactivity. However, the reduced reactivity of vanillin compared to 2-methoxy-4-vinylphenol suggests that the vinyl group may be more reactive than the aldehyde functionality on similar molecules. These compounds also show continued reactivity after HAA has been completely consumed, which indicates that these reactions do not occur solely due to phenol–formaldehyde type reactions. Although the GC-MS detectable fraction only accounts for a small overall mass percentage of the CFP oils, these data provide insight into potential reactions that drive thermal reactivity, and it is possible that the vinyl groups within larger lignin dimers and trimers also react to form high-molecular-weight compounds that contribute to increased viscosity.

**Figure 5:**
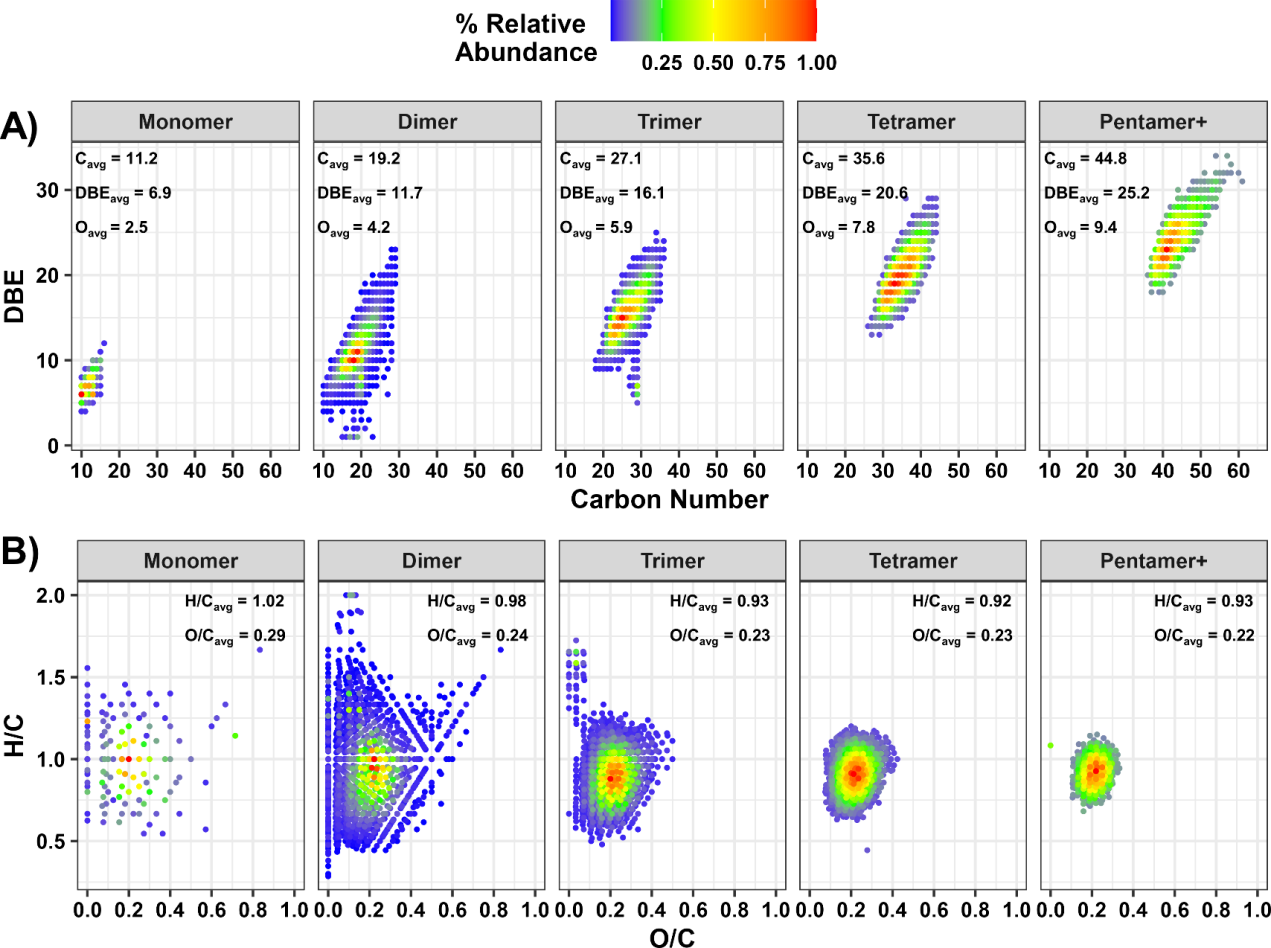
Positive-ion APCI FT-ICR MS derived plots for sample CFP-20O at 0 h of heating: (A) DBE vs carbon number for each region defined in Table S3. Weight-average carbon numbers, oxygen numbers, and DBE values are located in the top left corners. (B) van Krevelen plots for each oligomer region. Weight-average H/C and O/C values are located in the top right corners.

To complement the GC-MS data, FT-ICR MS was utilized to provide insight into the chemical composition changes in high-molecular-weight compounds formed during accelerated aging experiments. The mass spectra of the CFP oils revealed a multimodal distribution, which is consistent with the mass profile of lignin-derived oligomers (Figure S1). To further interpret these results, the data were divided into regions corresponding with the molecular weight ranges of the oligomer type (i.e., dimers, trimers, tetramers, and pentamers). Oligomer regions were divided by determination of the local minimum value between two distribution apexes. The boundaries used to estimate approximate dimer, trimer, and tetramer regions are provided in Table S3 with a representative mass spectrum shown in Figure S1. Figure 5A plots the carbon number vs double bond equivalents (DBE = C – (H/2) + (N/2) + 1), calculated from the FT-ICR MS data, for CFP-20O at the 0 h time point. The weight-average carbon number, DBE, and oxygen number values for each oligomer type are shown in the top left corner of each plot. An increase was observed in the carbon number and DBE ranges for each oligomer type, and the average increase in carbon number observed between regions was 8.4 carbon atoms per molecule. The average increase in DBE values was 4.6, and the average increase in oxygen number was 1.7 oxygen atoms per molecule. These values correspond well to lignin subunits.^38^ For example, 4-methyl guaiacol has a carbon number of 8 and a DBE value of 4 with 2 oxygen atoms, and 2-methoxy-4-vinylphenol (observed as highly reactive by GC-MS) has a carbon number of 9 with a DBE of 5 and 2 oxygen atoms. Both of these compounds are reasonable examples of lignin monomers that fit the average increases observed in Figure 5A and provide further support that oligomerization of lignin-derived compounds is a leading factor contributing to thermally driven molecular weight growth in CFP oil. The van Krevelen plots in Figure 5B indicate that the average H/C and O/C ratios for each region remain relatively consistent, which, in addition to the increases in carbon number, DBE, and oxygen number, indicates polymerization as the mechanism for the increased molecular weight. The average values for molar H/C and O/C have been previously reported for lignin as 0.7–1.1 and 0.3–0.4, respectively,^38^ which are in good agreement with the calculated values from FT-ICR MS here. It is worth noting that segmentation by mass, rather than by chemical formula boundaries, leads to the categorization of some compounds, especially in the dimer region, that falls outside of the compositional boundaries typically reported for lignin compounds. However, these compounds are in low abundance compared to the maximum at H/C = 1 and 0.2 and have only a small contribution to the trends discussed herein.

**Figure 6:**
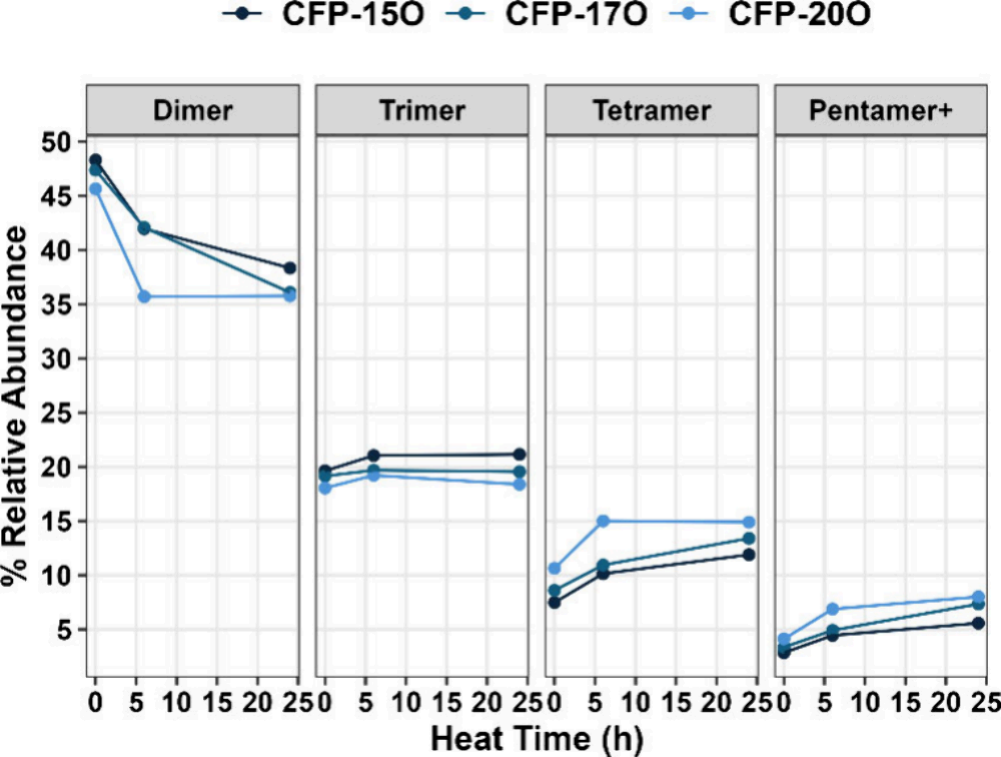
Percentage of the total FT-ICR MS signal that corresponds to the defined boundaries for dimer, trimer, tetramer, and pentamer+ mass regions. A description of the boundary regions is given in Table S3.

Figure 6 plots the summed percent relative abundance for each oligomer fraction as a function of time. The abundance of the dimer region decreases from 45–50% at 0 h to 35–40% after 24 h. A corresponding increase in the tetramer and pentamer+ regions indicates that larger oligomers are formed during aging. Interestingly, the abundance of the trimers remained relatively constant. This may be due to a high rate of self-condensation among dimer species to form tetramers directly or a steady-state condition in which the rate of trimer production is similar to the rate of trimer reaction.

**Figure 7:**
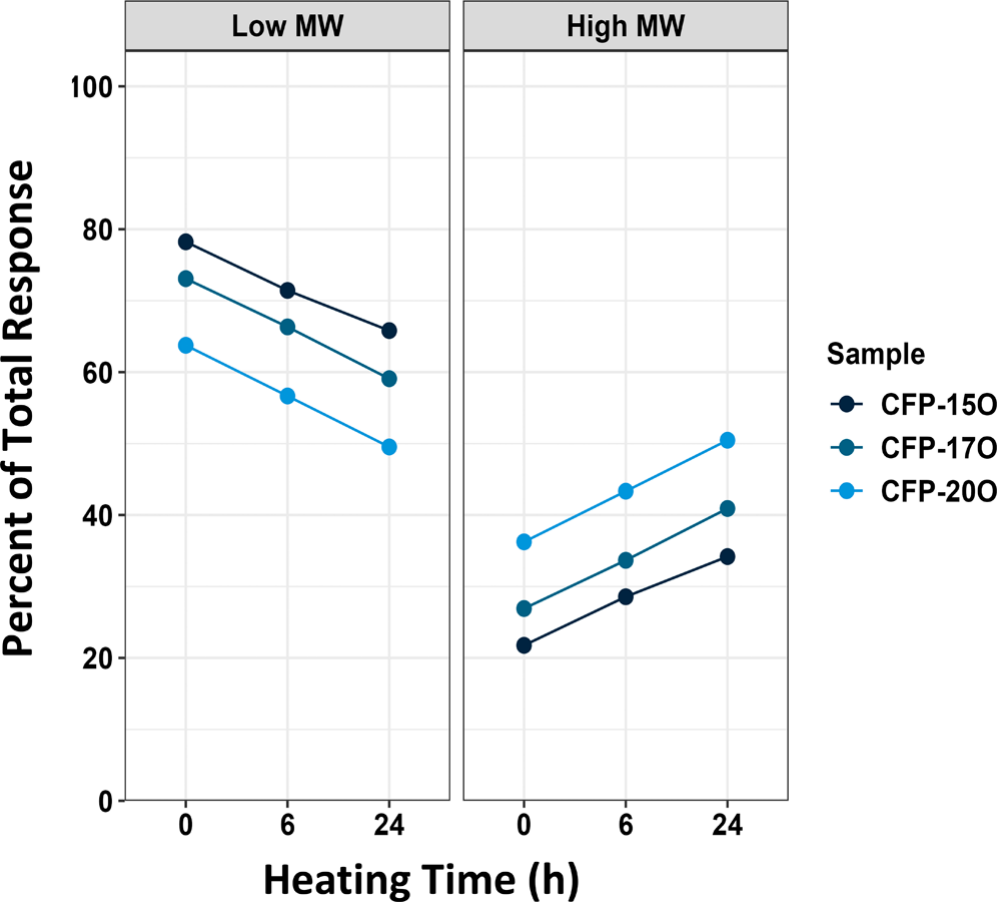
Integrated molecular weight regions from GPC analysis at three heating times: 0, 6, and 24 h of heating.

GPC analysis was performed to evaluate the apparent molecular weight distribution of the CFP oils before and after heating (Figure S2). These results revealed a clear thermally driven shift in the molecular weight distributions to higher molecular weights for all samples. To better understand the results, the GPC data were grouped into two regions consisting of compounds <500 Da and compounds >500 Da. The integrated area for each region was calculated, and the data are plotted in Figure 7. As expected, there is a positive correlation between heating time and the concentration of high molecular weight compounds, which is attributed to the coupling of reactive compounds. Figure 7 also indicates that CFP oils with higher oxygen content exhibited a higher relative percent of high-molecular-weight compounds (>500 Da). This observation highlights the potential of catalytic upgrading to improve the properties of CFP oil immediately upon production while also reducing the overall impact of reactivity on thermal aging during transportation, storage, and downstream processing.

**Figure 8:**
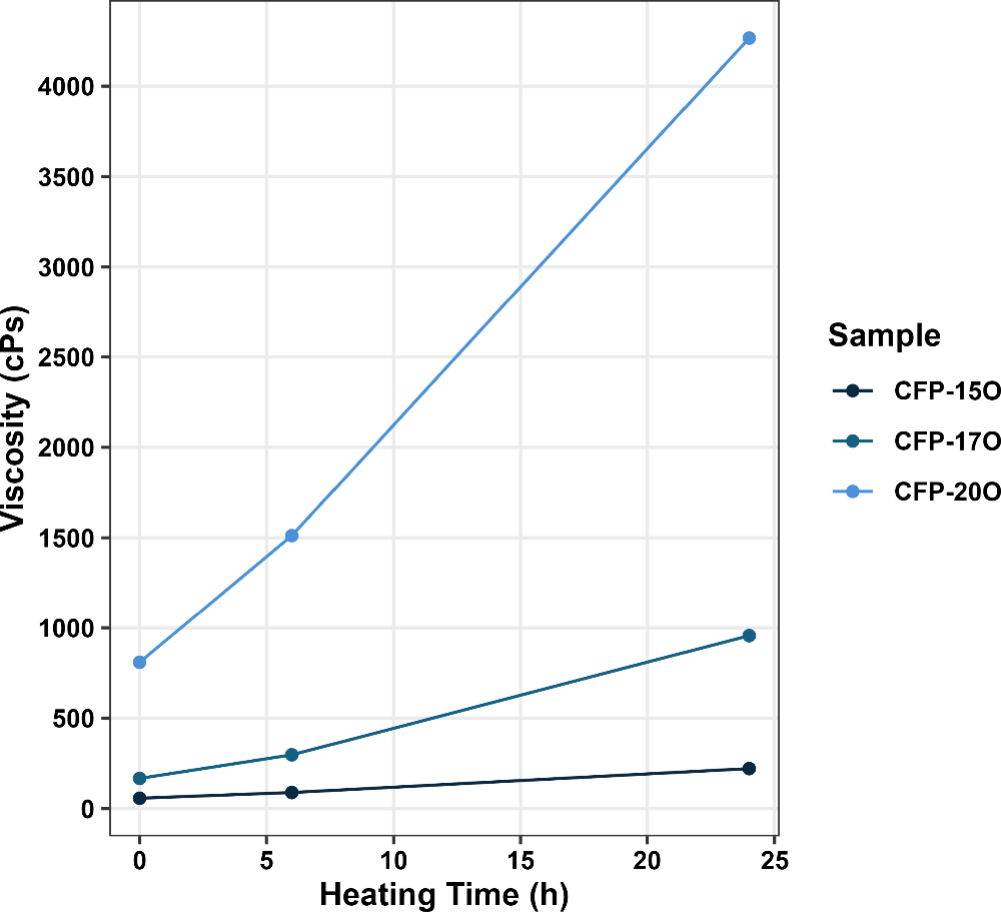
Viscosity of each CFP oil plotted as a function of heating time.

The viscosity of each CFP oil was measured after 0, 6, and 24 h of heating, and the data are given in Figure 8. Consistent with the GPC data, the results show an increase in viscosity as a function of heating time, and the CFP oils with a higher oxygen context exhibited a higher initial viscosity as well as a larger increase in viscosity upon heating. This observation provides further support that catalytic upgrading both improves the properties of the CFP oil immediately upon production and also stabilizes the oil against future thermal aging. Previous works for FP oils have indicated increases in viscosity upon aging of up to 50-fold.^9^ However, the overall viscosity of aged FP oils is generally less than 300 cPs due to the high percentage of water.^5,8,9,12^ The lower viscosity of FP oils compared to that of CFP oils is, however, misleading, as the high-molecular-weight lignin that forms in FP oils tends to precipitate due to the high water content, and phase separation has been reported for aged pyrolysis oils.^5,9^ Although the viscosity of aged CFP oil is high compared to many values reported for FP oil,^3,8,9,12,42^ aging experiments conducted by Mante and Agblevor for low-water-content FP oils (3–5% water) showed a viscosity increase for pine FP oil from approximately 100 to 1100 cPs after aging at room temperature for 6 months. FP oils derived from oak feedstocks showed a less pronounced increase from approximately 150 to 750 cPs after the same aging.^43^ These values are in line with the data reported for CFP oils and show a clear effect from the presence of water in FP oils. Notably, no phase separation or precipitation was observed for the CFP oils studied here, which reduces the risk of reactor plugging during upgrading.

### Reaction Chemistry of CFP Oils

**Scheme 1:**
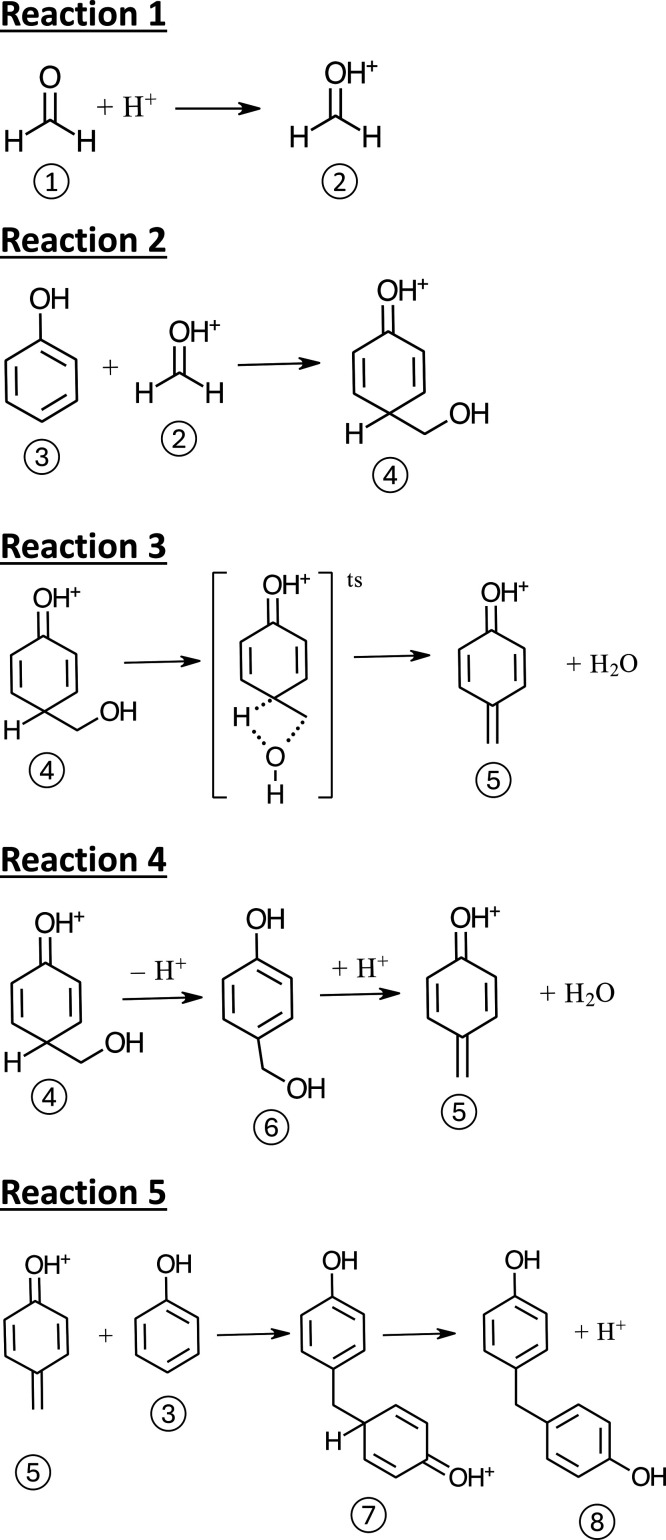
Mechanisms for Acid Catalyzed Phenol/Formaldehyde Reaction

The chemical complexity of CFP oil precludes the development of a comprehensive mechanism describing thermal reactivity. However, the analytical results presented above can be utilized to better understand the generalized reaction chemistry that underlies the process. For example, the consumption of carbonyls, anhydrosugars, and lignin-derived molecules observed by GC-MS (Figure 4) suggests that there are likely several reaction pathways that contribute to the formation of high-molecular-weight oligomers. The particularly high reactivity of HAA is consistent with proposed mechanisms, whereby aldehydes participate in condensation reactions to polymerize compounds such as phenols. An example is the acid catalyzed formation of phenol/formaldehyde resins. In this reaction (Scheme 1), a protonated aldehyde (**2**) adds to a phenolic compound (**3**), as shown in Reaction 2. The resulting protonated hydroxy methyl quinone (**4**) loses water by a direct intramolecular proton transfer (Reaction 3) or proton loss and acid catalyzed dehydration (Reaction 4) to form the resonance stabilized quinone methide (**5**). This intermediate can then combine with another phenol to form a dimer (**8**), as shown in Reaction 5. Acid titrations performed for select CFP oils are reported in Table S4 and range from 19 to 30 mg KOH/g. These values are consistent with previous measurements of CFP oils with similar oxygen contents^22^ and are considerably lower than the value of 76 mg KOH/g reported for the noncatalytic fast pyrolysis of pine.^23^ The comparatively low acidity of CFP oils may contribute to their improved thermal stability relative to noncatalytic fast pyrolysis by reducing the rates of phenol–formaldehyde coupling and other aldehyde condensation reactions. While this mechanism is proposed solely as a representative example, it is expected that these types of reactions occur across a wide variety of compounds to contribute to the thermally induced reactions that take place in CFP oils.

**Scheme 2:**
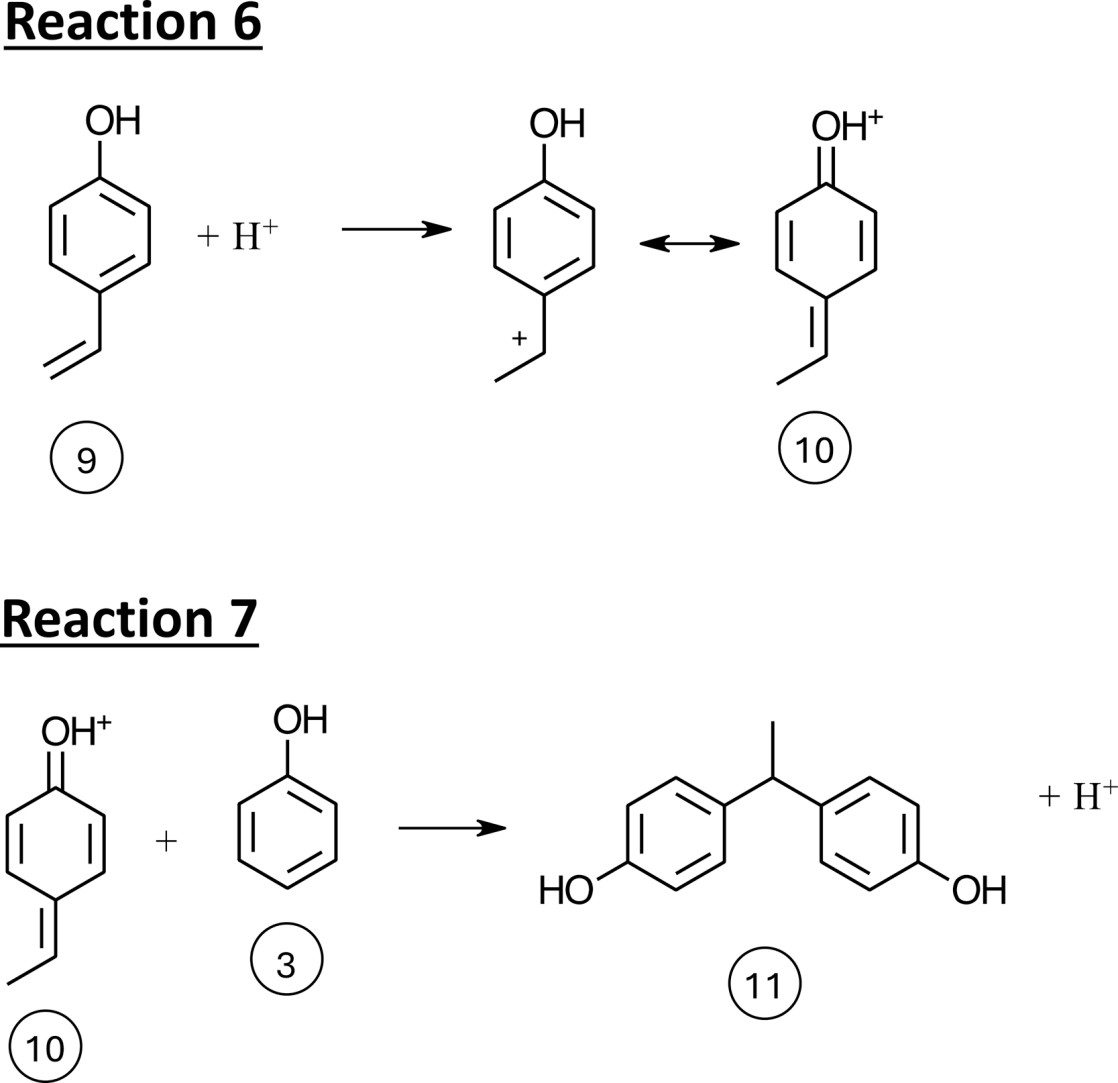
Mechanism for the Reaction of Protonated 4-Vinyl Phenol with Phenol through Resonance Stabilized Quinone Methide

In addition to short chain carbonyls such as HAA, vinylphenols were also rapidly consumed during the thermal stability experiments described in Figure 4. These compounds can be protonated to form quinone methides,^10^ and resonance stabilization of these intermediates has been shown to increase reactivity toward nucleophiles such as phenolic compounds. For instance, Scheme 2 shows that vinylphenol (**9**) can be protonated to form a quinone methide (**10**, Reaction 6) that may react with phenol to form a dimer (**11**) by electrophilic addition (Reaction 7). The nucleophilic centers on phenols are stabilized by resonance structures driven by OH groups, which are electron donating. These condensation reactions can lead to molecular growth and increased viscosity of the CFP oil without involving aldehydes or ketones, as evident by the fact that the consumption of vinyl-containing lignin compounds, as shown by GC-MS (Figure 4), continues even after HAA is fully consumed.

Additional condensation reactions may be possible over larger multi-ring compounds due to increased resonance stabilization of charge. This is supported by the comparatively high rate of consumption observed for 1-naphthalenol vs phenol during thermal reactivity experiments, as shown in Figure S3. In this plot, the phenol concentration decreases by 2–5% after heating at 100 °C for 24 h, whereas the 2-naphthalenol decreases by 30–40% over the same time period. This effect is consistent with the consumption of lignin dimers observed by FT-ICR MS (Figure 5) and attributed to increased resonance stabilization afforded by the extra aromatic ring (Figure S4).

### Impact of Catalytic Upgrading on Reactivity

The CFP oils utilized for the experiments described above were generated by using a single feedstock to minimize potentially confounding factors while gaining insight into the chemical and physical processes underlying thermal reactivity. Building on these results, an additional objective of this research was to determine the degree of catalytic upgrading that is needed to effectively stabilize the CFP oils and reduce the undesirable impacts of thermal reactivity on transportation, storage, and downstream processing. The importance of better understanding the relationship between catalytic upgrading and thermal reactivity is motivated by previous reports which have demonstrated that catalytic deoxygenation of pyrolysis vapors also results in undesirable carbon loss to coke and light gases.^23,44^ Thus, determining the necessary degree of catalytic deoxygenation provides an opportunity to effectively stabilize CFP oil while also maximizing the carbon efficiency of the integrated process. To help address this question, a series of accelerated aging experiments were performed using a variety of feedstocks and process conditions to produce CFP oils with oxygen contents ranging from 12 to 22 wt %, as described in Table 2.

**Table 2:** Select Feedstock Properties, Reaction Conditions, and Process Yields for CFP Oils

feedstock	ash content, wt %	B:C ratio	oil oxygen content, wt % dry basis	oil organic fraction mass yield, wt % dry biomass basis	oil aqueous fraction mass yield, wt % dry biomass basis
air classified pine	0.8	2.9	16	16	25
air classified pine	0.8	2.8	19	20	28
low ash pine	0.2	2.3	19	17	28
mid-range ash pine	1	2.8	22	21	27
high ash pine	1.3	1.7	14	12	27
high ash pine	1.3	3	20	15	26
forest residues	9.1	2.6	12	8	22

**Figure 9:**
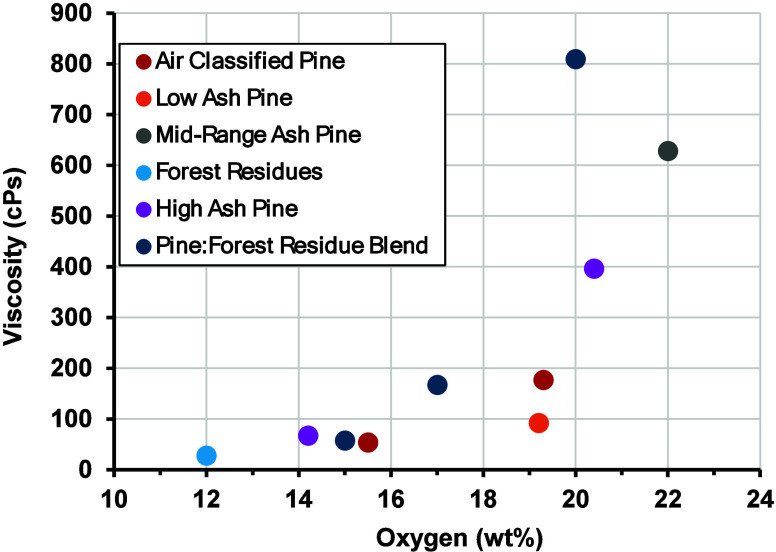
Viscosity as a function of oxygen content for as-prepared CFP oils.

For all samples, the as-prepared viscosity is plotted as a function of oil oxygen content in Figure 9. Viscosity is observed to increase gradually up to an oxygen content of 20 wt %, at which point the rate of viscosity increase accelerates for all feedstock material. Figures S2 and S5 show that the samples with an oxygen content of ≥20 wt % also have comparatively high molecular weight distributions as indicated by GPC analysis. The presence of this inflection point may be due to breakthrough of unreacted pyrolysis products at high B:C ratios or oligomerization of reactive species upon condensation of the CFP oils. Regardless, the results provide important insight into the degree of catalytic upgrading that is needed to stabilize the CFP oils and reduce the risk of undesired thermal reactions or plugging during downstream processing. Notably, the reactor system utilized to perform these experiments includes a hot gas filter upstream of the condensation unit, which mitigates the carryover of inorganic impurities into the CFP oils. To confirm the effectiveness of this approach, select CFP oils were evaluated using inductively coupled plasma optical emission spectroscopy (ICP-OES). These data are provided in Table S4 and indicate that the concentration of all measured elements was below detection limits with the exception of Si and S, which were present at <5 and <33 ppm, respectively. These elements are not expected to have a significant impact on the thermal stability of the CFP oils at the measured concentrations.

**Figure 10:**
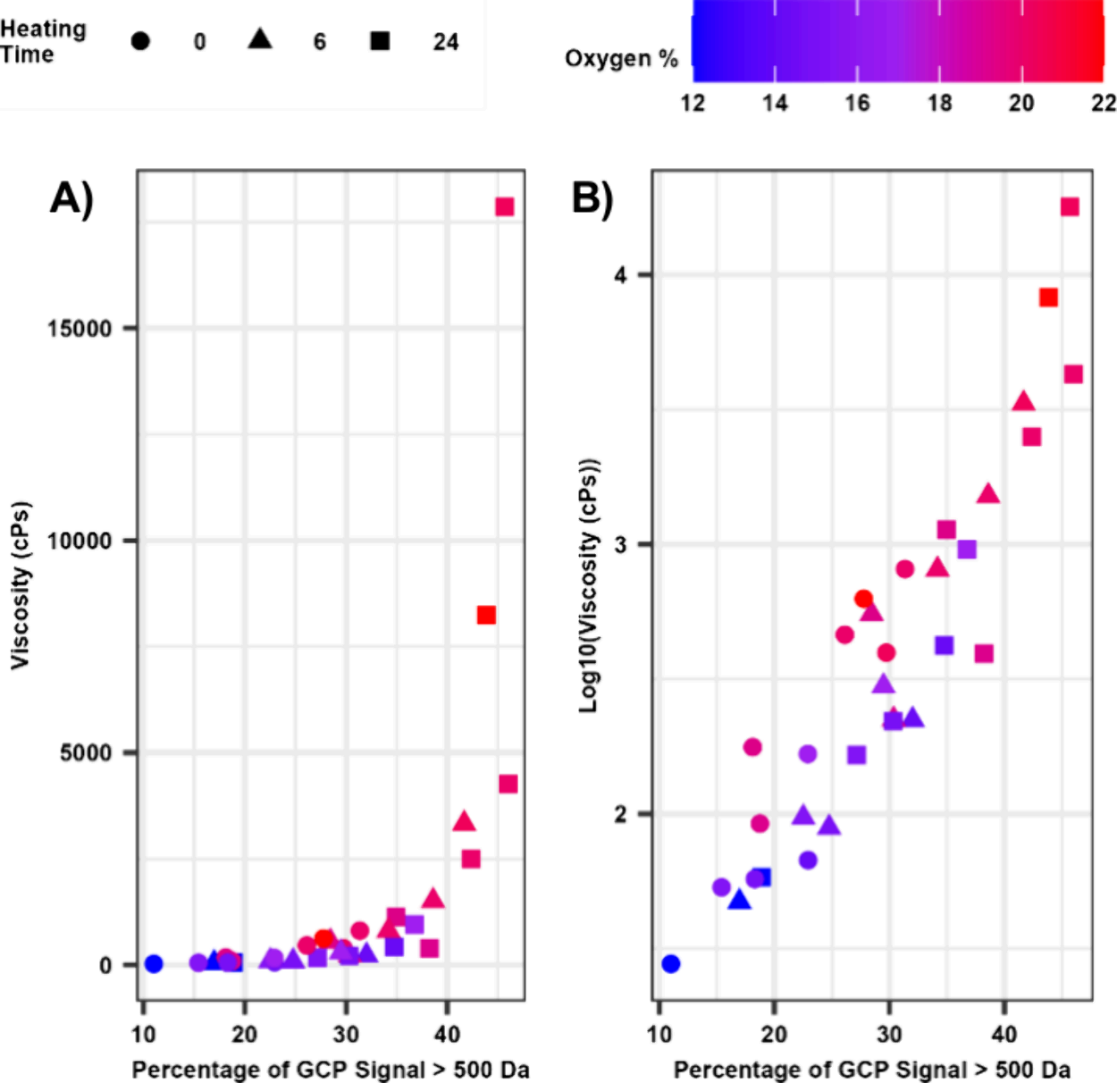
Viscosity plotted against the percentage of compounds with an apparent molecular weight of >500 Da using a (A) linear scale and (b) logarithmic scale. The marker shapes indicate the heating time, and the marker color indicates the oxygen content of the sample.

Data from the accelerated aging experiments reveal a clear relationship between the CFP oil viscosity and the percentage of compounds with an apparent molecular weight of >500 Da, as plotted in Figure 10. Previous reports have identified correlations between the average molecular weight and viscosity during aging of noncatalytic fast pyrolysis oils, as well as an increase in the pyrolytic lignin observed after aging.^3,4,8,10,45^ The results presented here suggest that the same trends existed for the CFP oils investigated in this study. As observed for the as-prepared samples (Figure 9), aging experiments confirm that the increase in viscosity and molecular weight accelerates as the oxygen content of the CFP oil approaches 20 wt %. This trend is also apparent in Figure S6, which plots the viscosity vs GPC data for each individual CFP oil. Acknowledging that the degree of catalytic upgrading for a specific process will be application specific, these collective data provide further support that an oil oxygen content of 20 wt % may represent an important threshold value that can be utilized to inform catalytic upgrading strategies and reduce the risk of process upsets during transportation, storage, and downstream conversion of CFP oils.

**Figure 11:**
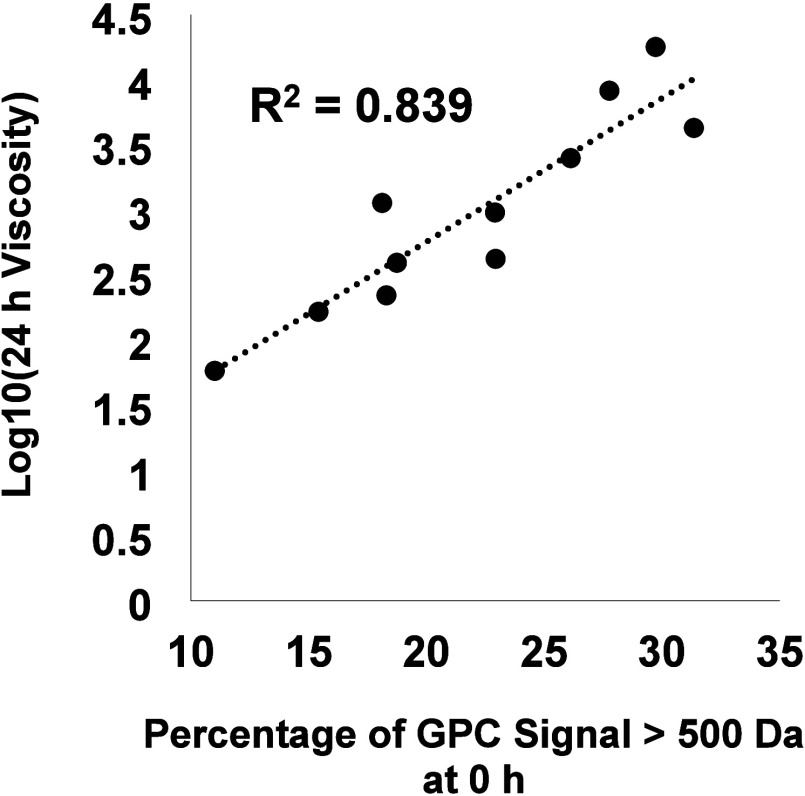
Viscosity after 24 h of heating plotted as a function of the percentage of compounds with an apparent molecular weight of >500 Da in the as-prepared CFP oils.

The predictive value of the high-molecular-weight fraction measured in the as-prepared samples (0 h) was also investigated. Figure 11 shows that the relationship between viscosity of the 24 h time point on a logarithmic scale is highly correlated with the percentage of compounds with an apparent molecular weight of >500 Da in the as-prepared oil, with an *R*^2^ value of 0.84. This correlation may be improved with additional insight into the structures and functional groups for high-molecular-weight compounds, and further analytical development is required to increase predictive capabilities. However, the results suggest that high-molecular-weight compounds in the initial oil may be predictive of thermally driven increases in viscosity, which would open opportunities to utilize widely available GPC methods to identify quality specifications and screen oil samples.

## Conclusions

In this research, accelerated aging experiments were coupled with comprehensive analytical characterization to deepen the mechanistic understanding of CFP oil reactivity and identify relationships between the degree of catalytic upgrading and thermally driven increases in viscosity and molecular weight. GC-MS and GPC results revealed that the coupling of short chain carbonyls, anhydrosugars, and lignin derivatives with conjugated vinyl groups plays an important role in thermally driven increases in viscosity and molecular weight. Complementary FT-ICR MS results suggested that similar reactions also likely occur in the heavy oil fraction, as evidenced by the apparent conversion of lignin dimers into higher-molecular-weight lignin oligomers. The reactivity of lignin monomers and dimers is further supported by chemical formula analysis that suggests polymerization of lignin compounds leads to an overall increase in carbon number and double bond equivalent (DBE) with stable H/C and O/C values. Collectively, these observations are consistent with reaction chemistries that proceed through a resonance stabilized protonated quinone methide intermediate that can be catalyzed by acids present in the CFP oils. Additional experiments were performed to determine the relationship between the degree of catalytic upgrading and thermally driven increases in viscosity across a wide range of biomass feedstocks. The results revealed an acceleration in viscosity increases as the oxygen content of the CFP oil approaches 20 wt %, which may represent an important threshold value that can be utilized to inform catalytic upgrading strategies and reduce the risk of process upsets during transportation, storage, and downstream conversion of CFP oils. Finally, the results suggest that the concentration of high-molecular-weight compounds in the as-prepared CFP oil may be predictive of thermally driven increases in viscosity, which would open opportunities to utilize widely available GPC methods to screen oil samples and avoid costly and disruptive process upsets. Future research needs include additional experiments performed across a wider range of feedstocks and CFP reaction conditions to validate the trends reported here. Further, accelerated aging experiments coupled with kinetic analysis and spiking of CFP oils with model compounds would provide valuable mechanistic information. Potential compounds of interest include reactive carbonyls such as hydroxyacetaldehyde, anhydrosugars such as levoglucosan, and lignin derivatives with conjugated vinyl groups such as 2-methoxy-4-vinylphenol. Kinetic experiments performed across a range of temperatures (e.g., 100–200 °C) could support the development of predictive models and the identification of deleterious compounds that could be targeted via catalytic upgrading. Finally, the development of application specific quality specifications for CFP oil are needed to derisk scale-up in the context of a specific process design or downstream use case.

## Supporting Information Available

Feedstock composition, compound groupings for GC-MS, boundaries used to estimate FT-ICR MS regions, acid titrations and elemental analysis of select CFP oils, and additional GPC and viscosity data from accelerated aging experiments (PDF)
