# Supplementary material for: Thermal Reactivity of Bio-Oil Produced from Catalytic Fast Pyrolysis of Biomass
Source: Energy Fuels. 2024 Oct 1;38(20):19626–38. doi: 10.1021/acs.energyfuels.4c03430 (PMC11492265; doi:10.1021/acs.energyfuels.4c03430)
Supplement: Supplementary file 1 — ef4c03430_si_001.pdf [file ef4c03430_si_001.pdf]

Thermal Reactivity of Bio-Oil Produced from Catalytic Fast Pyrolysis of Biomass

*Steven M. Rowland, Rianna Martinez, Cody J. Wrasman, Kristiina Iisa, Mark R. Nimlos, Michael B. Griffin**

National Renewable Energy Laboratory, 15013 Denver West Parkway, Golden, Colorado, 80401, United States

Table S1. Ultimate analysis of the feedstocks used to produce FP and CFP oils.

	Pine:Forest Residue Blend	Air Classified Pine	Mid-Range Ash Pine	High Ash Pine	Low Ash Pine	Forest Residues
C	49.10%	51.60%	50.90%	49.20%	50.10%	47.50%
H	6.20%	7.30%	6.30%	6.50%	6.30%	5.80%
N	0.20%	0.20%	0.20%	0.10%	0.10%	0.40%
H_2O	3.20%	2.30%	2.00%	4.20%	1.40%	5.30%
Ash	1.10%	0.78%	1.00%	1.30%	0.22%	9.10%
O_{dry} (by diff)	41.90%	39.00%	40.70%	40.90%	42.60%	34.00%

Table S2. Molecules comprising each compound grouping as identified by GCMS and reported in Figure 1. The concentration profile during accelerated aging experiments for molecules shown in bold is reported in Figure 4.

**Aromatics**		
Benzene, propyl-	Styrene	Naphthalene, 1-methyl-
Benzene, 1-ethyl-4-methyl-	Toluene	Naphthalene, 1-ethyl-
Benzene, 1-ethyl-2-methyl-	Ethylbenzene	Naphthalene, 1,3-dimethyl-
Benzene, 1-ethyl-3-methyl-	Benzene, 1,3-dimethyl-	Naphthalene, 2,3-dimethyl-
Benzene, 1,2,4-trimethyl-	o-Xylene	Naphthalene, 2,7-dimethyl-
Benzene, 2-propenyl-	p-Xylene	Naphthalene, 2-(1-methylethyl)-
Benzene, 1-ethenyl-3-methyl-	Indane	Fluorene
Benzene, 1-ethenyl-4-ethyl-	Indene	Phenanthrene
1H-Indene, 2,3-dihydro-5-methyl-	Mesitylene	Retene
Benzene, 1-ethenyl-4-methyl-	1H-Indene, 1-methyl-	Anthracene, 9-methyl-

Benzene, 1-butynyl-	2-Methylindene	Phenanthrene, 2-methyl-
Benzene, (2-methyl-1-propenyl)-	Benzene, 1-propynyl-	Phenanthrene, 1-methyl-
Benzene, 1-propenyl-	Indan, 1-methyl-	Phenanthrene, 3,6-dimethyl-
Benzene, 1-methyl-4-propyl-	1H-Indene, 3-methyl-	Phenanthrene, 2,3,5-trimethyl-
Benzene, 4-ethyl-1,2-dimethyl-	Naphthalene	Naphthalene, 2-methyl-
Benzene		
**Acids**		
Acetic acid	Acetic acid, (acetyloxy)-	Propanoic acid
Butanoic acid		
**Carbonyls**		
**Acetaldehyde, hydroxy-**	2-Cyclopenten-1-one, 2-methyl-	Butyrolactone
**2-Butenal**	2-Cyclopenten-1-one, 3-methyl-	1H-Inden-1-one, 2,3-dihydro-
2-Cyclopenten-1-one, 2-hydroxy-3-methyl-	Succindialdehyde	Cyclohexanone
Heptanal	2-Propanone, 1-(acetyloxy)-	3-Buten-2-one, 3-methyl-
2-Cyclopenten-1-one, 2,3-dimethyl-	2-Butenal, (E)-	2,3-Pentanedione
2-Pentanone, 4-hydroxy-4-methyl-	Methyl vinyl ketone	3-Penten-2-one
1,2-Cyclopentanedione	2,3-Butanedione	Acetoin
1,2-Cyclopentanedione, 3-methyl-	2-Butanone	1-Hydroxy-2-butanone
Cyclopentanone, 3-methyl-	2-Propanone, 1-hydroxy-	2-Cyclohexen-1-one
3-Methylcyclopentane-1,2-dione	Cyclopentanone	2-Cyclopenten-1-one
**Furans**		
**Furfural**	5-Hydroxymethylfurfural	Furan, 2,5-dimethyl-
**2-Furancarboxaldehyde, 5-methyl-**	Ethanone, 1-(2-furanyl)-	2-Vinylfuran
2(5H)-Furanone	Benzofuran	Furan, 2-methyl-
2(5H)-Furanone, 3-methyl-	Benzofuran, 2-methyl-	Furan, 2,5-dibutyl-
4-Methyl-5H-furan-2-one		
**Phenols**		
Benzaldehyde, 3-hydroxy-	Hydroquinone	Homovanillic acid
Phenol, 2-methoxy-4-(1-propenyl)-, (Z)-	Phenol, 2-methoxy-	Phenol, 2,6-dimethyl-
Phenol, 4-ethyl-	Creosol	**Vanillin**
Phenol, 3-ethyl-	Phenol, 4-ethyl-2-methoxy-	Apocynin
2-Propanone, 1-(4-hydroxy-3-methoxyphenyl)-	**2-Methoxy-4-vinylphenol**	Phenol, 3,4-dimethyl-
Phenol, 2-methoxy-3-(2-propenyl)-	4-vinylphenol	**Coniferyl aldehyde**
Phenol, 2-ethyl-5-methyl-	Eugenol	Phenol, 2-methyl-
Phenol, 3-ethyl-5-methyl-	**trans-Isoeugenol**	p-Cresol
Phenol, 2,3,6-trimethyl-	coniferyl alcohol	Phenol, 3-methyl-
Phenol, 3-ethyl-4-methyl-	Phenol, 4-propyl-	Phenol
Phenol, 2-methoxy-6-(2-propenyl)-	Phenol, 2-methoxy-4-propyl-	Phenol, 2,3-dimethyl-
**Sugars**		
Levoglucosan		

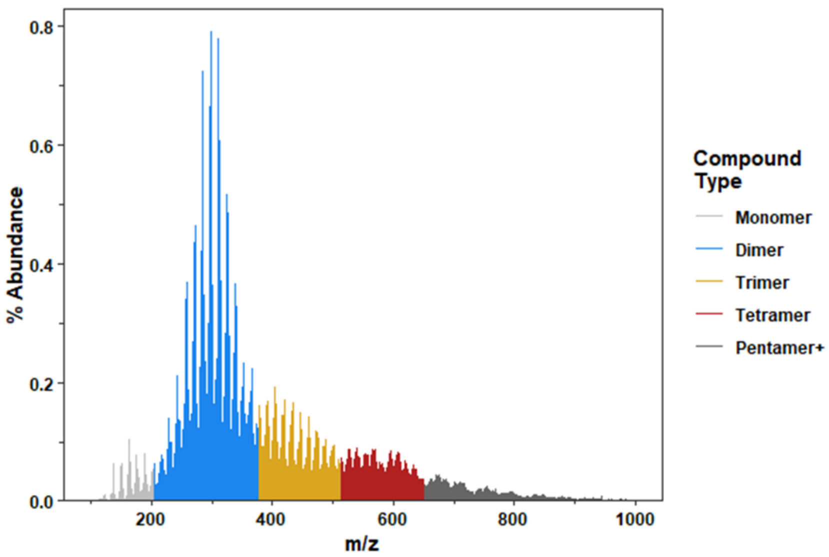

Figure S1. Positive-ion APCI FT-ICR MS spectral peaks that correspond to the boundaries described in Table S3.

Table S3. Boundaries used to estimate approximate dimer, trimer, and tetramer classification.

Oligomer Type	m/z Range
**Monomer**	100-205
**Dimer**	205-377
**Trimer**	377-513
**Tetramer**	513-650
**Pentamer+**	> 650

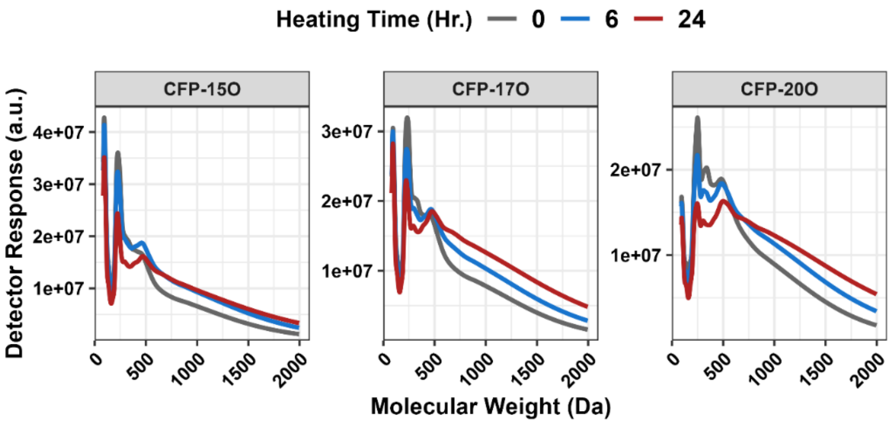

Figure S2. GPC chromatograms, normalized to total signal, for CFP oils from aging experiments at 0, 6, and 24 h time points.

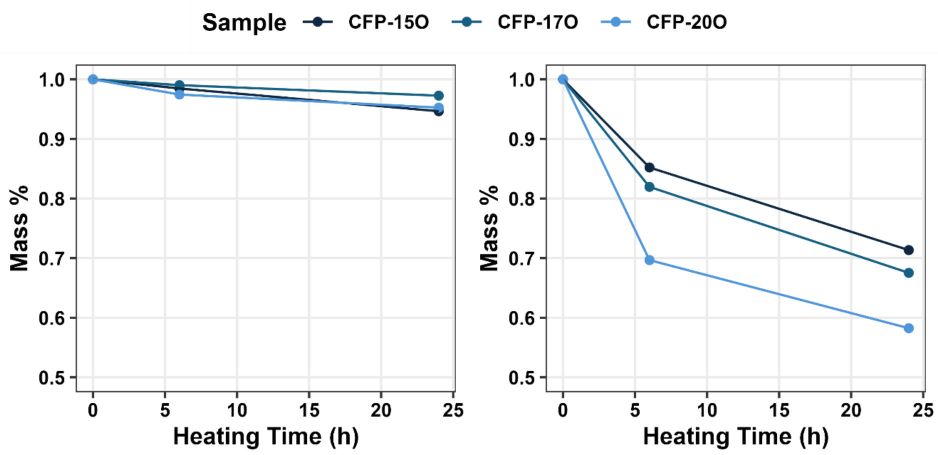

Figure S3. Reactivity plots for phenol (left) and 2-naphthalenol (right) shows the increased reactivity with increased ring stabilization of protonated intermediates.

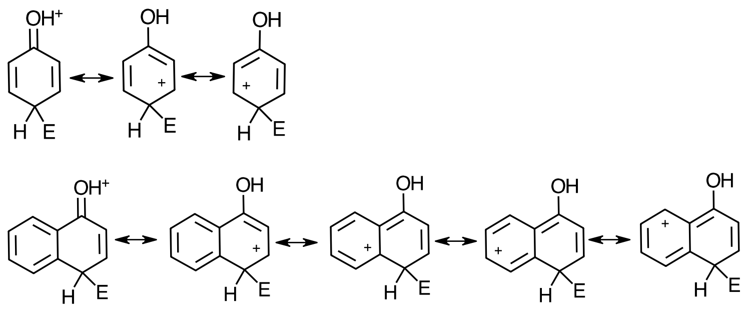

E = Electrophile

Figure S4. An example of the greater resonance stabilization affored by the extra aromic ring in 1-naphthalenol compared to phenol.

Table S4. Acid titration and ICP-OES data for select CFP-oils

Feedstock	Pine:Forest Residue Blend	Pine:Forest Residue Blend	Mid-Range Ash Pine
Oil Oxygen Content , wt% dry basis	17	20	22
Carboxylic Acid Number, mg KOH/g	19	24	30
Ag, ppm	< 3	< 3	< 3
Al, ppm	< 3	< 3	< 3
B, ppm	< 3	< 3	< 3
Ca, ppm	< 3	< 3	< 3
Cd, ppm	< 3	< 3	< 3
Co, ppm	< 3	< 3	< 3
Cr, ppm	< 3	< 3	< 3
Cu, ppm	< 3	< 3	< 3
Fe, ppm	< 3	< 3	< 3
Ga, ppm	< 3	< 3	< 3
K, ppm	< 3	< 3	< 3
Li, ppm	< 5	< 5	< 5
Mg, ppm	< 3	< 3	< 3
Mn, ppm	< 3	< 3	< 3
Na, ppm	< 3	< 3	< 3
Ni, ppm	< 3	< 3	< 3
P, ppm	< 5	< 5	< 5
Pb, ppm	< 3	< 3	< 3
Pt, ppm	< 3	< 3	< 3
S, ppm	31.5	32.4	26.9
Si, ppm	4.8	< 3	< 3
Sr, ppm	< 3	< 3	< 3
Ti, ppm	<15	<15	<15
Zn, ppm	< 3	< 3	< 3

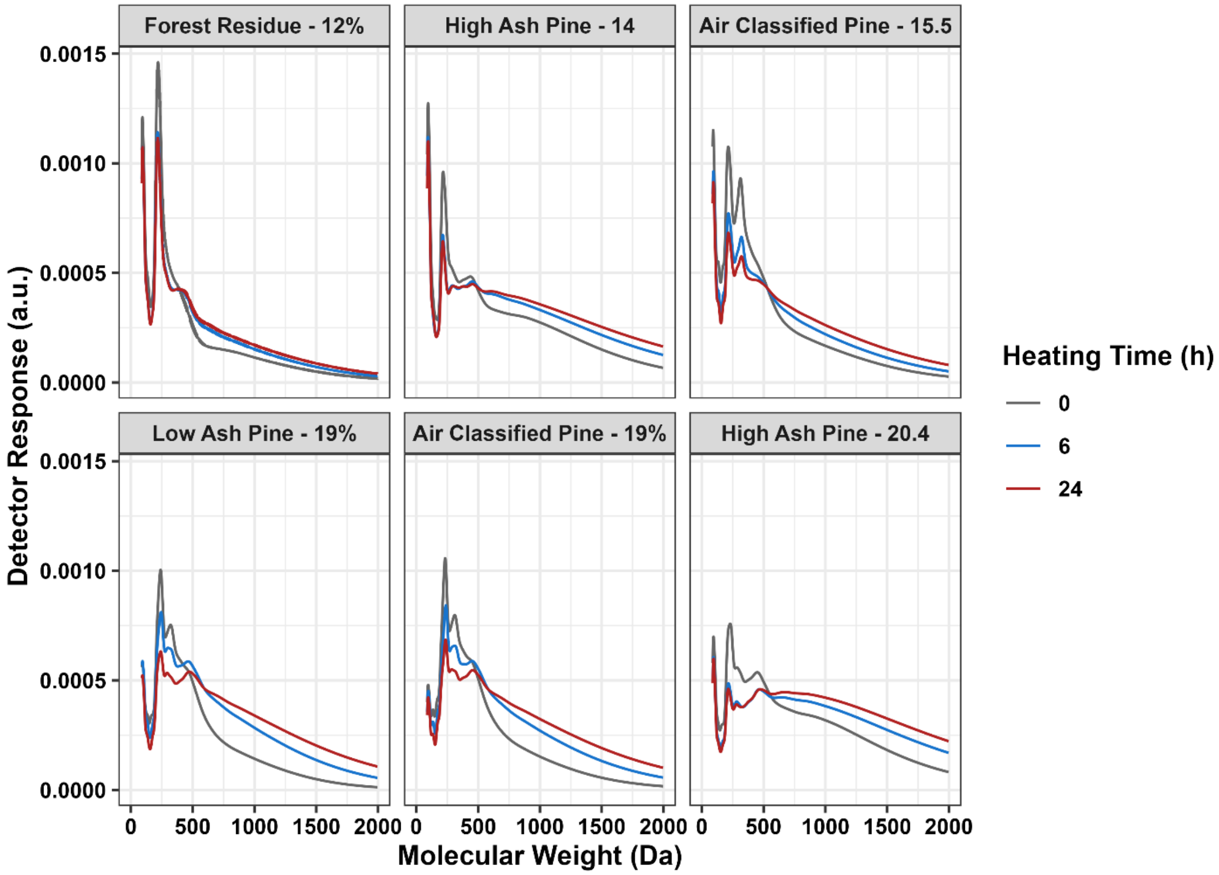

Figure S5. GPC chromatograms, normalized to total signal, for CFP oils in Table 2 from aging experiments at 0, 6, and 24 h time points.

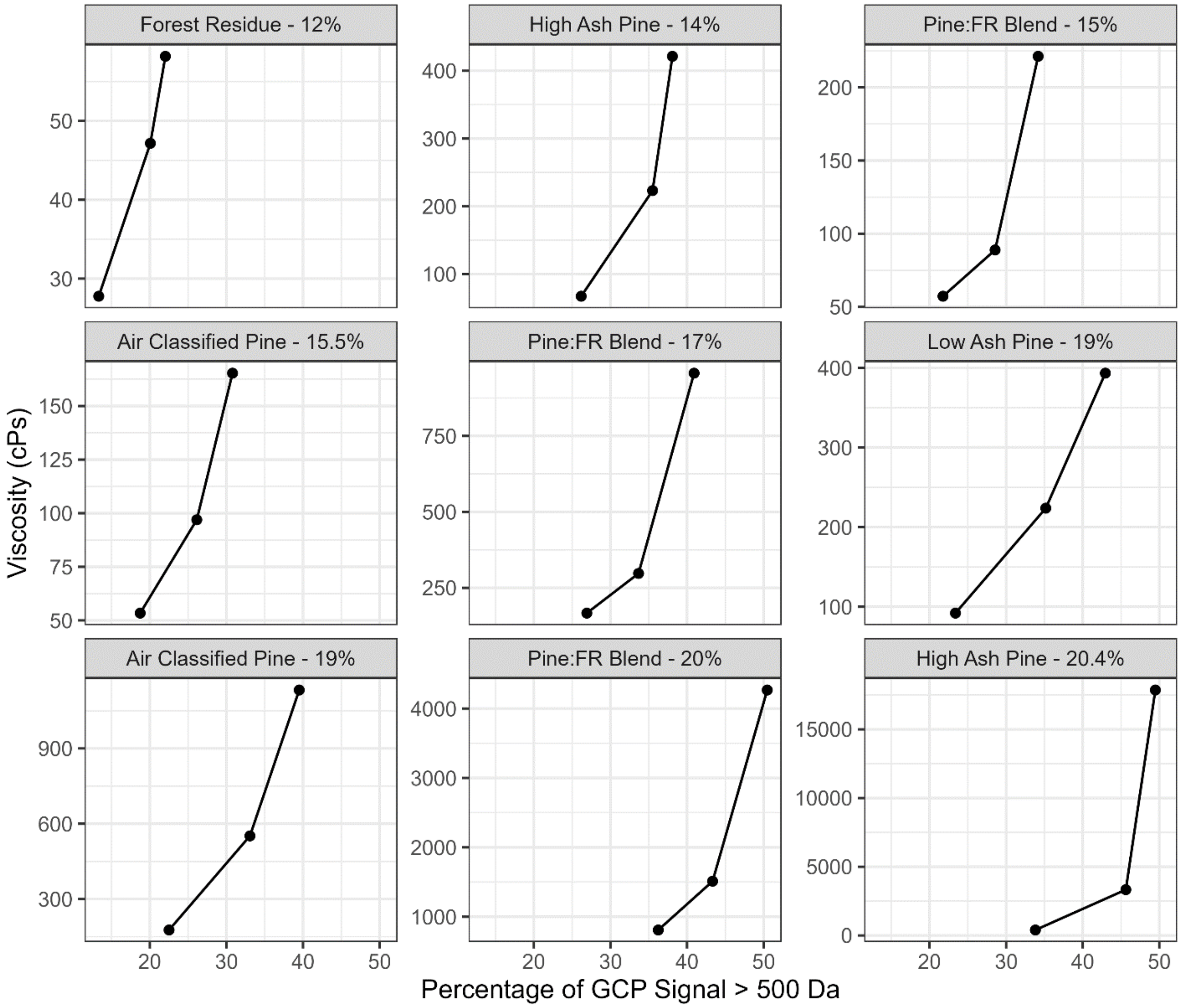

Figure S6. The viscosity for each sample from Table 1 and Table 2 plotted vs. the percentage of GPC signal that corresponds to compounds with masses greater than 500 Da. The data is separated here by each oil to show deeper insight into the correlation between high molecular weight compounds and viscosity.
